# Association of retroperitoneal lymphangioleiomyomatosis with endosalpingiosis: a case report

**DOI:** 10.4076/1757-1626-2-6331

**Published:** 2009-08-07

**Authors:** José Manuel Lorente Herce, Virgilio Ruiz Luque, José Aguilar Luque, Pablo Martínez García, Daniel Díaz Gómez

**Affiliations:** 1 Department of General and Gastrointestinal Surgery, Hospital Universitario de ValmeSevilleSpain; 2 Department of General and Gastrointestinal Surgery, Hospital San Juan de DiosSevilleSpain

## Abstract

Lymphangioleiomyomatosis is a rare disorder of unknown origin that usually presents pulmonary symptoms. Retroperitoneal lymphangioleiomyomatosis without lung involvement has rarely been reported. We present a 38-year-old woman, the fourth case reported of retroperitoneal lymphangioleiomyomatosis with endosalpingiosis in the literature.

## Introduction

Lymphangioleiomyomatosis (LAM) is a rare disorder of unknown origin that almost exclusively affects women of childbearing age [1,2]. It is characterized by the formation of thin-walled cysts and hamartomatous proliferation of immature smooth muscle cells of the lymph nodes, lymphatics, blood vessels, alveoli, mediastinum, and retroperitoneum [2].

Pulmonary LAM is the most common form of occurrence of this disorder. Occasionally, pulmonary LAM is presented with extrapulmonary symptoms characterized by retroperitoneal lympadenopathy, chylous ascites, and renal angiomyolipomas (AML) [2,3]. However, extrapulmonary LAM without lung involvement has rarely been reported. In this study, we present the fourth case of retroperitoneal LAM with endosalpingiosis reported in the literature.

## Case presentation

**Figure 1.**
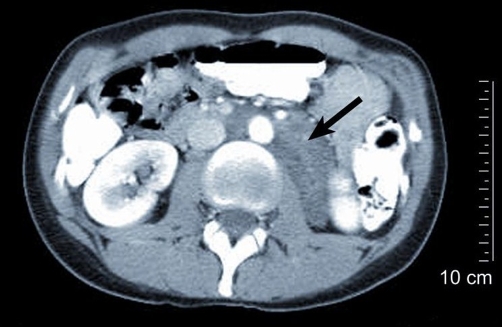
CT scan of the abdomen showing a retroperitoneal mass (arrow).

**Figure 2.**
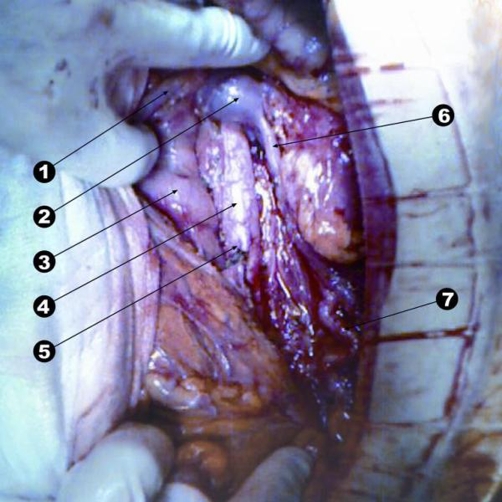
Surgical image after excision of the mass. (1) Inferior mesenteric vein. (2) Left renal vein. (3) Duodenum. (4) Abdominal aorta. (5) Inferior mesenteric artery. (6) Left lower polar vein. (7) Ureter.

A 38-year-old white woman with a history of oral contraceptive use, presented with a non-quantified weight loss over a 9-month period with no further clinical complaints. Furthermore, laboratory data showed no abnormalities. Computed tomography (CT) studies of the abdomen demonstrated a 10-cm cystic mass in the retroperitoneum, surrounding the aorta, on the medial side of the inferior vena cava bordering the superior and inferior mesenteric arteries, the left renal hilum, pancreatic body, and left psoas muscle (Figure 1). Under radiological suspicion of retroperitoneal lymphangioma, the decision was made to excise the cystic mass as well as the periaortic and retroperitoneal lymphadenopathy (Figure 2). The postoperative period was uneventful, and the patient was released 5 days after the operation.

Histopathologic study of the specimen demonstrated LAM and endosalpingiosis in the retroperitoneal lymph nodes.

So far, the patient has been asymptomatic, with normal CT and normal pulmonary function tests (spirometry, plethysmography, and diffusion capacity).

## Discussion

The LAM is a rare entity of unknown etiology that occurs in women, normally in their reproductive years, and can be associated with Tuberous Sclerosis (TS). The prevalence of sporadic LAM is around one per million in the United Kingdom, France, and the United States [2,3].

The pathogenesis of the disease is still unknown. It has been observed that about 25% of the patients who underwent treatment for LAM previously had oral contraceptives. However, a national case control study found no association between oral contraceptive use and LAM [4].

There are very few reported cases of extrapulmonary LAM without pulmonary affectation, and Kebria identified 21 cases [5].

The coexistence of proliferation of atypical smooth muscle cells with endosalpingiosis in the retroperitoneal lymph nodes in patients with LAM is a unusual finding. Endosalpingiosis is a benign condition characterized by the presence of glands lined by ciliated tubal-type epithelium [2].

Prevalence of extrapulmonary LAM with endosalpingiosis is unknown and there are only three cases reported in the literature [1,6]. Matsui *et al.* [1] described two women, 36 and 50-year-old, respectively, without previous pulmonary symptoms. In both the cases, a cystic mass was found in the retroperitoneum; the first during a cesarean and the other one during a study of unspecified abdominal pain. Fukunaga *et al*. [6] reported a third case of extrapulmonary LAM associated with endosalpingiosis in a 25-year-old woman. This patient did not have any pulmonary affectation, as the aforementioned case.

Clinical manifestations of extrapulmonary LAM are usually rare. Abdominal pain is an unusual symptom.

Abdominal imaging by ultrasound may show renal AML and retroperitoneal adenopathy [3] with cystic degeneration which tends to conglomerate and appear like a retroperitoneal cystic tumor. However, a differential diagnosis is necessary for other tumor-like retroperitoneal lymphangioma.

Treatment of pulmonary LAM is based on hormonal manipulations to prevent progressive lung destruction. At present, early therapy with intramuscular or oral medroxyprogesterone is the most-accepted treatment [7]. Moreover, a symptomatic treatment with a thoracostomy tube or chemical pleurodesis is recommended for chylous effusion, while a paracentesis or peritoneovenous shunt is recommended for chylous ascites. Patients with progressive deterioration of pulmonary function can benefit from lung transplantation, presenting a forecast similar to transplant patients of other complications [8]. However, few options remain in managing the retroperitoneal LAM with no other location. The lack of diagnostic suspicion in most cases and the need for differential diagnosis of other entities make surgical resection as the most commonly used strategy.

As Matsui *et al.* described, many of patients with extrapulmonary LAM develop pulmonary LAM within 1-2 years after diagnosis [9]. Various reports describe a poor prognosis when lungs are involved, with progression and death from pulmonary complications within 10 years of diagnosis [2].

Owing to the uncertain behavior of this entity, the complete removal of the tumor and adjacent lymph is recommended to prevent expansion and consequent clinical compression. The frequency of malignant LAM cases associated with endosalpingiosis has not yet been described. Given the rarity of its presentation, the possibility of malignant degeneration cannot be disregarded.

An excision of the cystic mass and lymphadenectomy was performed. During the surgical intervention, it was noted that the cystic mass was located in the retroperitoneal left center-lateral region surrounding the aorta, the space between the vena cava and aorta, and renal pelvis, adhering to the brink of the lateral lumbar vertebral bodies and the front edge of the left psoas. Despite the structures involved, excision was performed to conserve them and continue with an extensive lymphadenectomy.

In summary any patient diagnosed with retroperitoneal LAM must be studied and followed up to make an early diagnosis of a possible pulmonary involvement. This facilitates the treatment of the patients in the early stages of the disease, thus improving the prognosis. In addition, surgery is the only therapeutic option in most of these cases.

## Abbreviations

CT: computed tomography
LAM: lymphangioleiomyomatosis
TS: tuberous sclerosis
